# Supplementary material for: First Identification, Recombinant Production, and Structural Characterization of a Putative Structural Protein from the Haseki Tick Virus Polyprotein
Source: Biomolecules. 2025 Dec 3;15(12):1690. doi: 10.3390/biom15121690 (PMC12730655; doi:10.3390/biom15121690)
Supplement: Supplementary file 1 [file biomolecules-15-01690-s001.zip › biomolecules-3973405-supplementary.pdf]

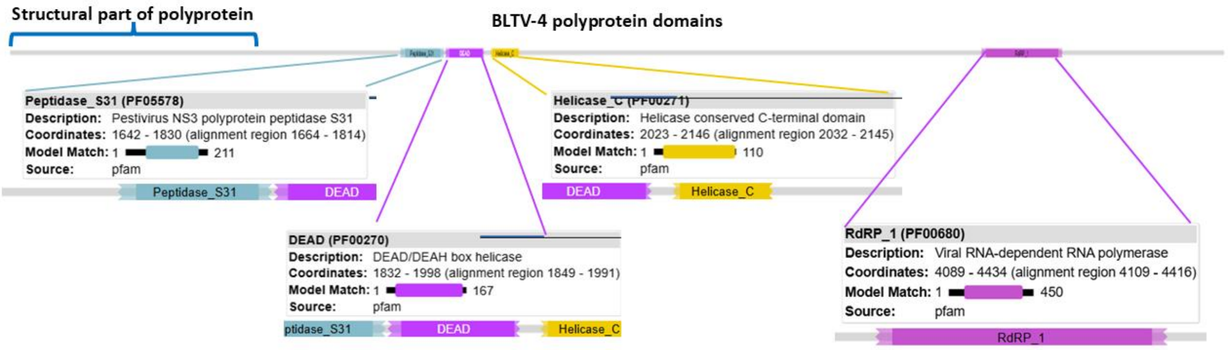

Figure S1. Domain structure of Bole tick virus 4.

Table S1. Oligonucleotides used to synthetically assemble a DNA copy of a HSTV SP1-coding region.

Oligonucleotide length (position in HSTV polyprotein)	Oligonucleotide name	Oligonucleotide sequence
60 (1-60)	1HSTV_F	ATGCCGCGCTGGCTGGCCCAACAAGGTTACAACT ACTCGTTTCATGGTGTGTGGAATATC
60 (40-99)	1HSTV_R	GAAACCTTCCGGCTCGCAGCCTGGCTGTGGACTT GGGGGGATATTCCACACACCATGAAA
60 (80-139)	2HSTV_F	GCTGCGAGCCGGAAGGTTTCGACAGTTGGGTTAA AGACAGATGGCGTAGCCTCCTGTGGT
60 (120-179)	2HSTV_R	AGGCGACAACACGTGTGGTTGGTGGCAGGCGAT GAATCGAACCACAGGAGGCTACGCCAT
60 (160-219)	3HSTV_F	AACCACACGTGTTGTCGCCTACAGCGCCATGAGT ATTTAAAGCACGCTTGTCGTGCTCAG
60 (200-259)	3HSTV_R	GTGCGCGATCCACGGATGCCAGATAATCCGCCAG GGCAACCTGAGCACGACAAGCGTGCT
37 (240-277)	4HSTV_F	GGCATCCGTGGATCGCGCACCGACGCCGGCGACA GCG
60 (257-316)	4HSTV_R	TTACCGCTTCATCAAAGCGGCATTGACGCGCACA GAACGGCGCTGTCGCCGGCGTCGGTG
60 (296-355)	5HSTV_F	GCCGCTTTGATGAAGCGGTAAACGTCGTACAGGA AGCAGTCGATGCCGGCAGCTGGCACC
60 (335-394)	5HSTV_R	GATACCACGCATTTTTACGGCACCCCACAATGTC AGTCGGGTGCCAGCTGCCGGCATCGA
60 (375-434)	6HSTV_F	CCGTAAAAATGCGTGGTATCGGTTTGGCGATCAT CTGGTTCCACAGCCCTGCTTGCCGGA
60 (415-474)	6HSTV_R	CGGAGCCACTAAACGGCTCGGATCAGGGGACTGT CGAGGGTCCGGCAAGCAGGGCTGTGG
48 (454-501)	7HSTV_F	CCGAGCCGTTTAGTGGCTCCGACCGGATCTGGGC TTGGCGCAGCCAAA

Table S2. Predicted transmembrane and cytoplasmic/extramembrane domains within the putative structural region (residues 1–1250) of the HSTV polyprotein. Domain boundaries were determined by consensus prediction using TMHMM, CCTOP, and PredictProtein. Domain boundaries are also visualized in Fig. 1; this table provides numerical annotation.

HSTV membrane topology domains	HSTV domain positions, a.a.	HSTV domain length, a.a.
Extramembrane	1-173	173
Transmembrane	174-189	16
Cytoplasmic	190-651	462
Transmembrane	652-671	20
Extramembrane	672-831	160
Transmembrane	832-851	20
Cytoplasmic	852-1250	399

Table S3. Structural comparison of HSTV SP1 with the pestiviral Erns and Npro based on pairwise structural alignment.

PDB ID	Name of virus	TM-score	RMSD, Å	Length, a.a.	Aligned Residues, a.a.
	**Erns**				
4DVK	BVDV-1	0.69	3.51	158	120
4DW3	BVDV-1	0.63	3.61	154	111
4DWA	BVDV-1	0.63	3.49	157	115
4DWC	BVDV-1	0.62	3.49	156	113
4DVL	BVDV-1	0.61	3.57	165	112
4DVN	BVDV-1	0.61	3.61	154	110
4DW4	BVDV-1	0.61	3.54	157	113
4DW5	BVDV-1	0.64	3.29	161	120
4DW7	BVDV-1	0.62	3.55	161	113
	**Npro**				
3ZFP	BVDV-3 (strain D32/00_HoBi)	0.27	6.08	148	38
3ZFN	BVDV-3 (strain D32/00_HoBi)	0.28	6.01	148	41
3ZFQ	BVDV-3 (strain D32/00_HoBi)	0.26	5.8	148	40
3ZFT	BVDV-3 (strain D32/00_HoBi)	0.28	6.14	148	38
3ZFR	BVDV-3 (strain D32/00_HoBi)	0.25	5.66	149	41
3ZFO	BVDV-3 (strain D32/00_HoBi)	0.27	6.06	164	40
3ZFU	BVDV-3 (strain D32/00_HoBi)	0.27	5.28	164	45
4H9K	CSFV	0.29	6.09	154	38
4H9J	CSFV	0.25	5.86	154	37

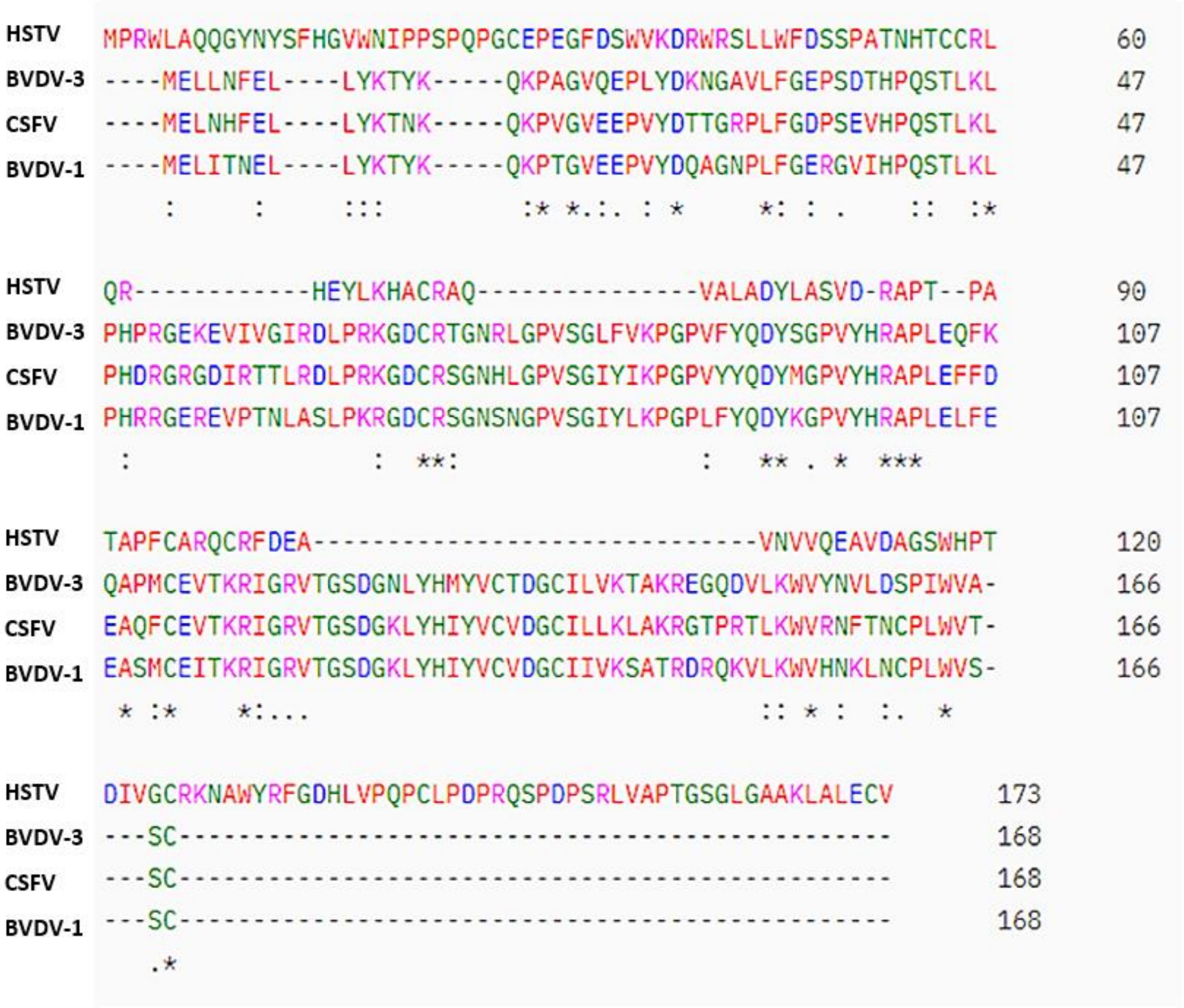

Figure S2. Multiple sequence alignment of HSTV SP1 with Npro from representative *Pestivirus* genus.

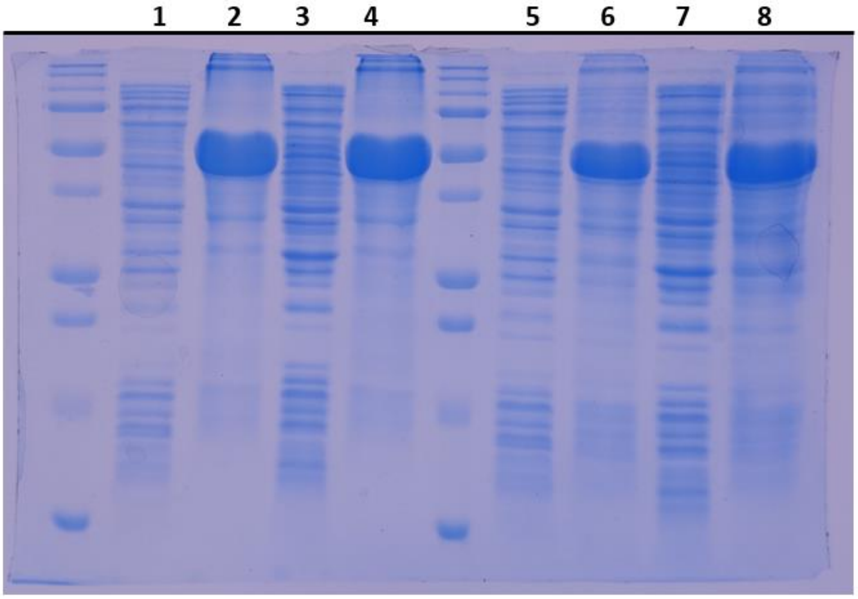

Figure S3. SDS-PAGE of total protein lysates from BL21(DE3)/SP1-PS-mNeonGreen and KRX/SP1-PS-mNeonGreen producer strains following induced expression.

- Lane 1 and lane 5: BL21(DE3)/SP1-PS-mNeonGreen uninduced control.
- Lane 2 and lane 6: BL21(DE3)/SP1-PS-mNeonGreen cells harvested after 20 h of induction.
- Lane 3 and lane 7: KRX/SP1-PS-mNeonGreen uninduced control.
- Lane 4 and lane 8: KRX/SP1-PS-mNeonGreen cells harvested after 20 h of induction.

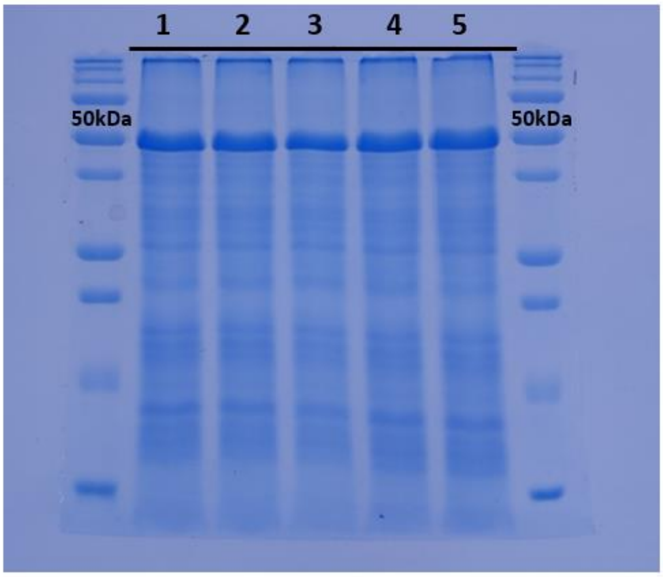

Figure S4. SDS-PAGE of total protein lysates and purified fractions from BL21(DE3)/SP1-PS-mNeonGreen cultured under varying zinc conditions. Lanes: 1 – standard conditions; 2 – +1 mM $ZnSO_4$; 3 – +10 mM $ZnSO_4$; 4 – purified protein with 1 mM $ZnSO_4$; 5 – purified protein with 10 mM $ZnSO_4$.

Table S4. *E. coli* producer strains used for recombinant HSTV SP1 protein expression. “+” – strain, construct combinations that were experimentally tested in this study; “–” - combinations that were not attempted.

**Genetic construction**	***E. coli* strains**				
	BL-21(DE3)	KRX	HyperStable	T7EvoPro	LysTech
pET-HSTV-SP1	+	+	-	-	-
pEASY-HSTV-SP1	+	+	+	-	-
14x-His-Tev-SP1	+	+	+	+	+

Table S5. Conditions and outcomes of induced cultivation of recombinant HSTV SP1-producing *E. coli* strains. Green: soluble HSTV SP1 expression; yellow: insoluble (inclusion body) HSTV SP1 expression; red: no detectable HSTV SP1 expression. “+” - expression conditions (strain + induction parameters) yielding detectable levels of the target recombinant protein (either in soluble fraction or inclusion bodies); “–” - conditions under which no recombinant protein was detected by SDS–PAGE.

***E. coli* producer strains**	**Cultivation temperature, °C**						**IPTG, mM**
	37	32	28	25	20	16	

BL-21(DE3)/pET-HSTV-SP1	+	+	+	+	+	+	1
KRX/pET-HSTV-SP1	-	+	+	+	+	-	1
BL-21(DE3)-pEASY/HSTV-SP1	-	-	-	-	-	-	1
	-	-	-	-	-	-	1,5
	-	-	-	-	-	-	2
KRX/pEASY-HSTV-SP1	-	-	-	-	-	-	1
	-	-	-	-	-	-	1,5
	-	-	-	-	-	-	2
HyperStable/pEASY-HSTV-SP1	-	-	-	-	-	-	1
	-	-	-	-	-	-	1,5
	-	-	-	-	-	-	2
BL-21(DE3)/14x-His-Tev-SP1	+	+	+	+	+	+	1
	+	+	+	+	+	+	0,5
KRX/14x-His-Tev-SP1	+	+	+	+	+	+	1
	+	+	+	+	+	+	0,5
HyperStable/14x-His-Tev-SP1	+	+	+	+	+	+	1
	+	+	+	+	+	+	0,5
T7-EvoPro/14x-His-Tev-SP1	+	+	+	+	+	+	1
	+	+	+	+	+	+	0,5
LysTech/14x-His-Tev-SP1	+	+	+	+	+	+	1
	+	+	+	+	+	+	0,5

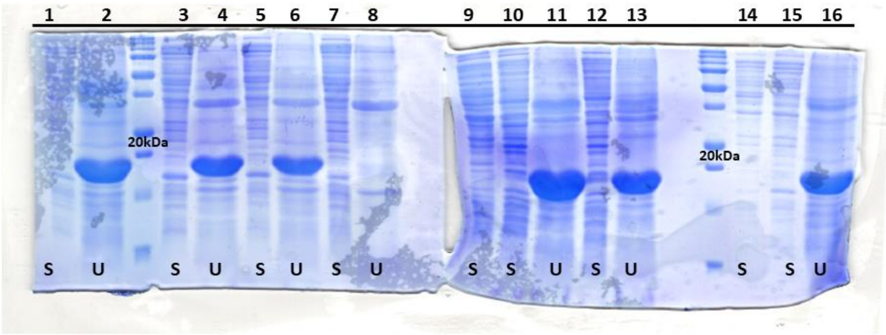

Figure S5. SDS-PAGE analysis of recombinant HSTV SP1 expression in *E. coli* strains carrying the 14×His-TEV-SP1 construct. Odd-numbered lanes correspond to the soluble (S) fraction; even-numbered lanes correspond to the inclusion body fraction (U). The expected molecular weight of the chimeric SP1 protein is ~19 kDa.

- Lanes 1–2: BL21(DE3)/14×His-TEV-SP1;
- Lanes 3–4: KRX/14×His-TEV-SP1;
- Lanes 5–8: T7-EvoPro/14×His-TEV-SP1 (lanes 5–6: 1 mM IPTG; lanes 7–8: 0.5 mM IPTG);
- Lanes 9–11: HyperStable/14×His-TEV-SP1 (lanes 9 and 10: soluble replicates);
- Lanes 12–16: LysTech/14×His-TEV-SP1 (lanes 12–13: 1 mM IPTG; lanes 14–16: 0.5 mM IPTG, with lane 15 as a soluble replicate).

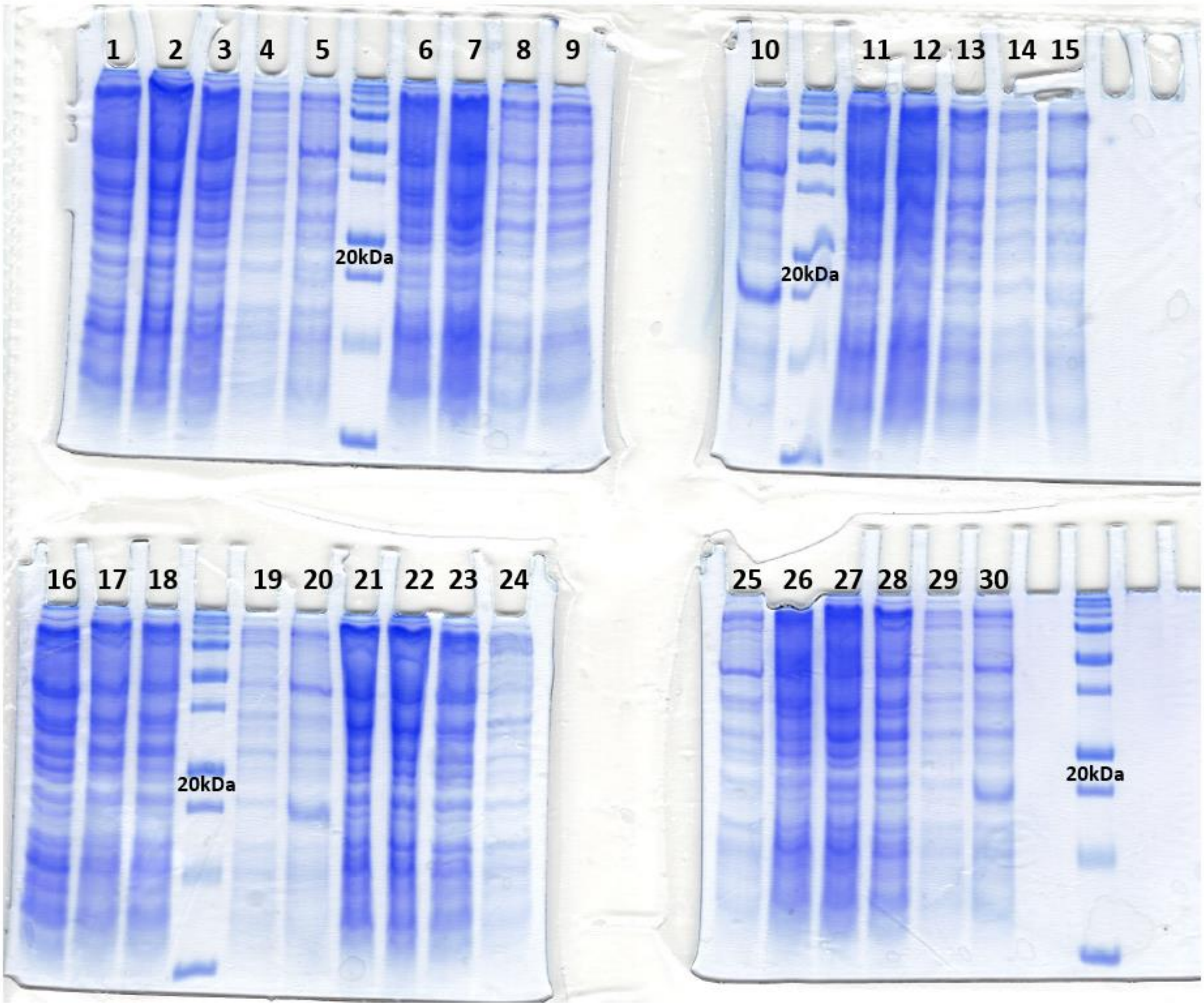

Figure S6. SDS-PAGE analysis of recombinant HSTV SP1 expression in *E. coli* strains carrying the pEASY-HSTV-SP1 construct. No detectable expression of the expected ~20.8 kDa chimeric HSTV SP1 protein was observed under any tested condition.

- Lanes 1–5: BL21(DE3)/pEASY-HSTV-SP1 induced with 1.5 mM IPTG at 37, 30, 25, 20, and 16 °C, respectively.
- Lanes 6–10: KRX/pEASY-HSTV-SP1 induced with 1.5 mM IPTG at 37–16 °C.
- Lanes 11–15: KRX/pEASY-HSTV-SP1 induced with 1.0 mM IPTG at 37–16 °C.
- Lanes 16–20: HyperStable/pEASY-HSTV-SP1 induced with 1.0 mM IPTG at 37–16 °C.
- Lanes 21–25: HyperStable/pEASY-HSTV-SP1 induced with 0.5 mM IPTG at 37–16 °C.
- Lanes 26–30: HyperStable/pEASY-HSTV-SP1 induced with 1.5 mM IPTG at 37–16 °C.

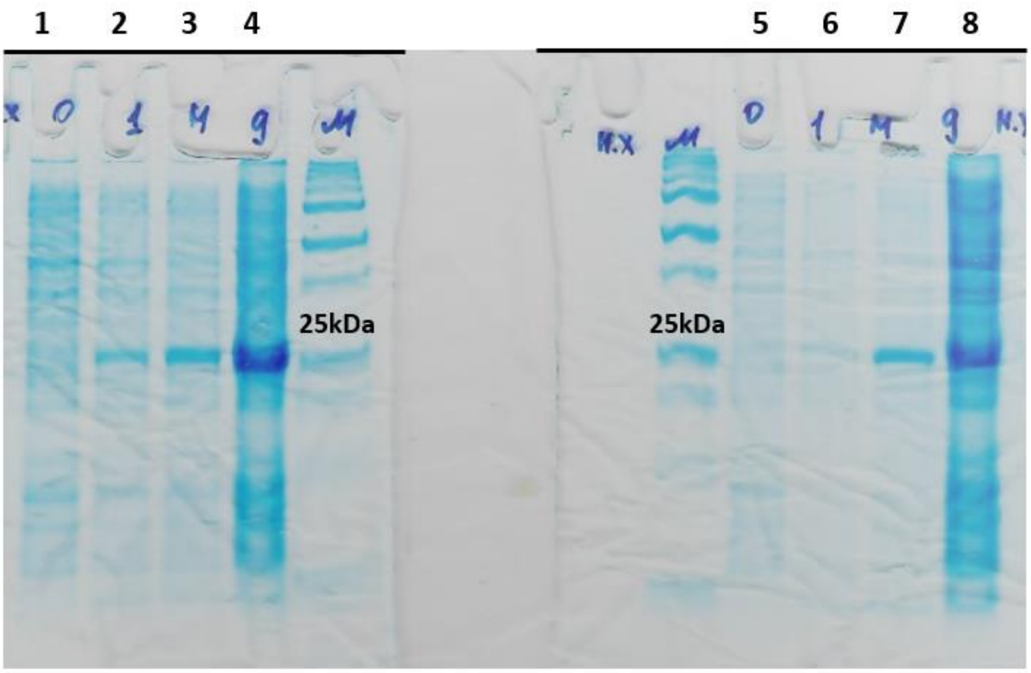

Figure S7. SDS-PAGE analysis of recombinant HSTV SP1 expression in *E. coli* strains carrying the pET-HSTV-SP1 construct. The expected molecular weight of the chimeric HSTV SP1 protein is ~26 kDa.

- Lanes 1–4: BL21(DE3)/pET-HSTV-SP1 harvested at 0, 1, 5, and 20 hours post-induction, respectively.
- Lanes 5–8: KRX/pET-HSTV-SP1 harvested at 0, 1, 5, and 20 hours post-induction, respectively.

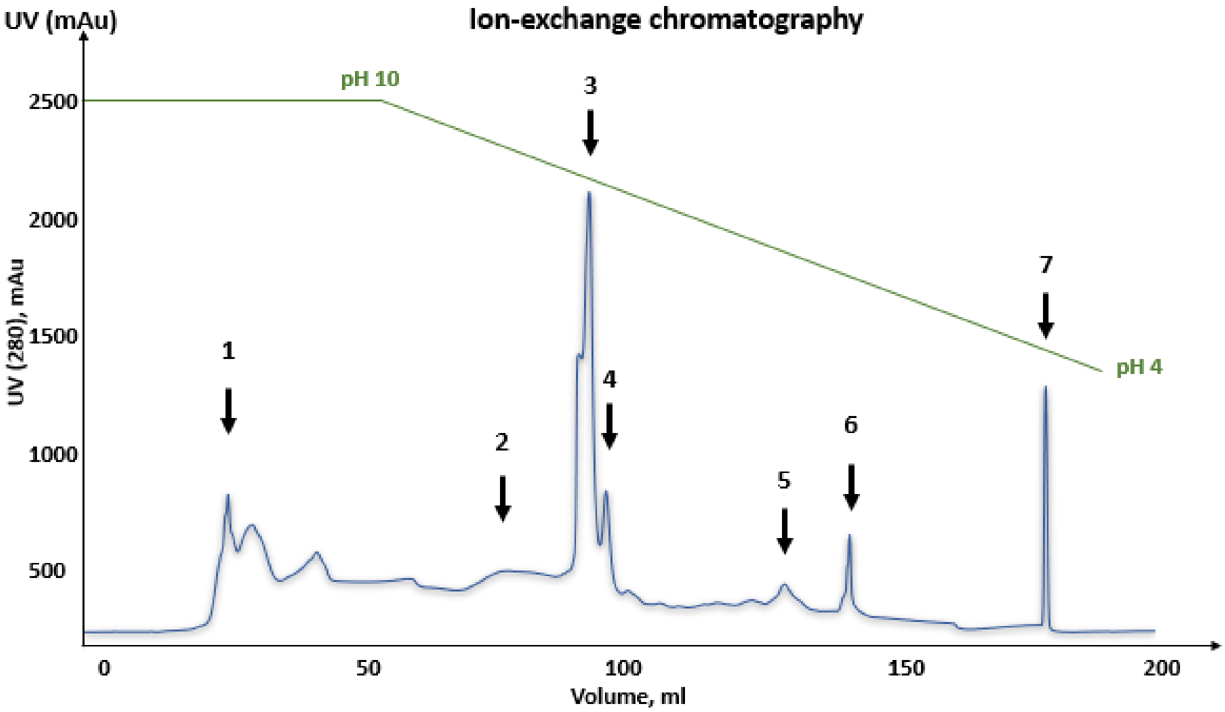

(a)

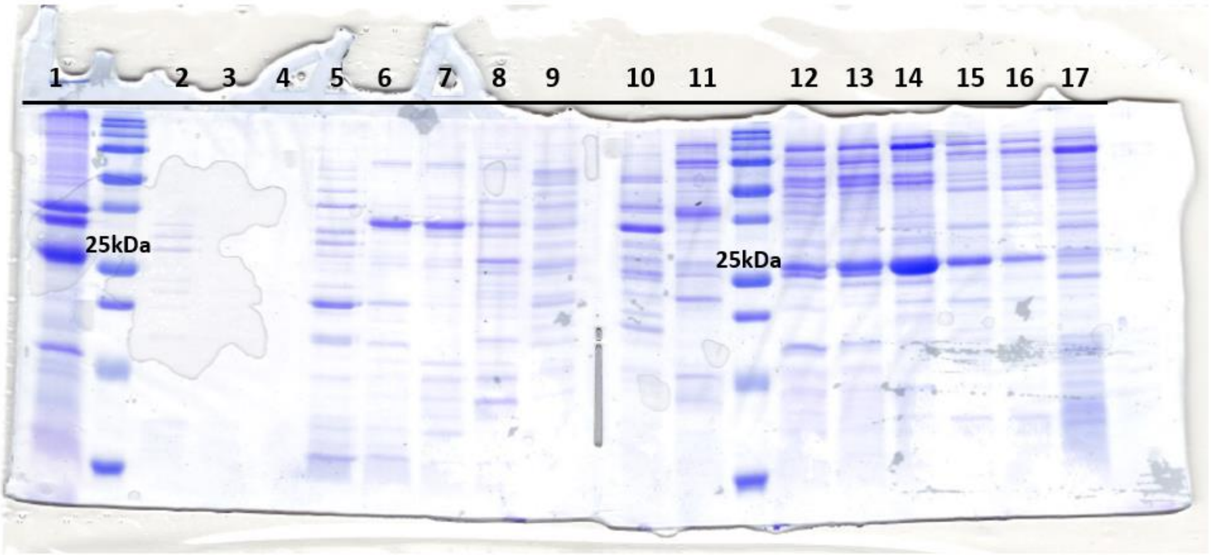

(b)

Figure S8. Ion-exchange chromatography of recombinant HSTV SP1. **(a)** Chromatogram showing seven elution peaks (labeled 1–7). The target HSTV SP1 protein (~26 kDa) eluted primarily in peak 5. **(b)** SDS-PAGE analysis of collected fractions:

- Lane 1 – total cell lysate;
- Lanes 2–5 – peak 1;
- Lanes 6–8 – peak 2;
- Lanes 9–10 – peak 3;
- Lane 11 – peak 4;
- Lanes 12–15 – peak 5;
- Lane 16 – peak 6;
- Lane 17 – peak 7.

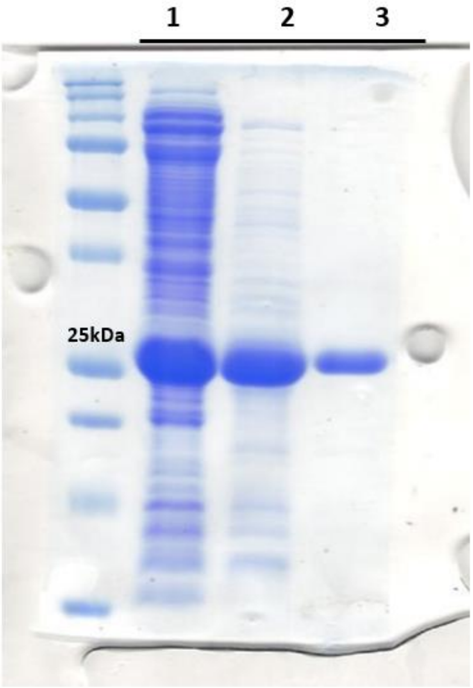

Figure S9. SDS-PAGE analysis of HSTV SP1 purification at successive chromatographic steps. The expected molecular weight of HSTV SP1 is ~26 kDa; purity exceeds 95% in the final fraction.

- Lane 1: total cell lysate;
- Lane 2: HSTV SP1 fraction after ion-exchange chromatography;
- Lane 3: purified HSTV SP1 after size-exclusion chromatography.

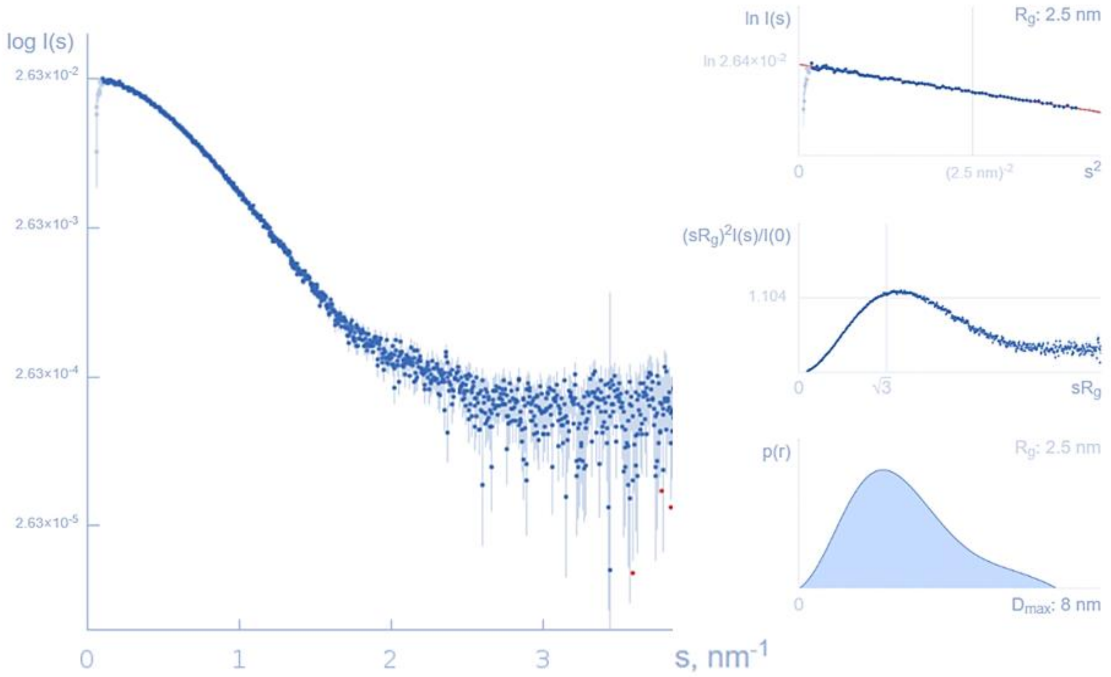

Figure S10. SAXS profile of HSTV SP1 at 3.12 mg/mL: log I(s) versus scattering vector magnitude s (nm^{-1}), reflecting particle shape and size in solution. Diagnostic plots are shown on the right: (i) Guinier region used to estimate the radius of gyration (Rg) and forward scattering intensity I(0); (ii) dimensionless Kratky plot for assessing compactness and flexibility; and (iii) pair-distance distribution function P(r), yielding Rg and the maximum particle dimension (Dmax) in real space.

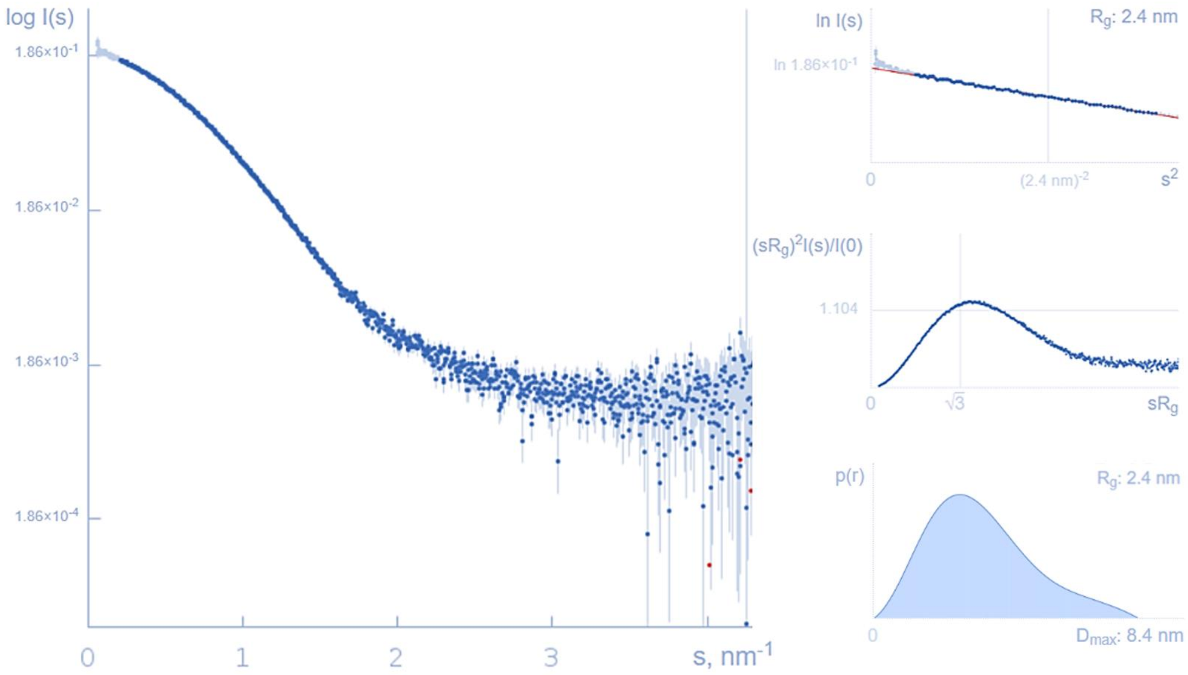

Figure S11. SAXS profile of HSTV SP1 at 12.31 mg/mL: log I(s) versus scattering vector magnitude s (nm^{-1}), reflecting particle shape and size in solution. Diagnostic plots are shown on the right: (i) Guinier region used to estimate the radius of gyration (Rg) and forward scattering intensity I(0); (ii) dimensionless Kratky plot for assessing compactness and flexibility; and (iii) pair-distance distribution function P(r), yielding Rg and the maximum particle dimension (Dmax) in real space.

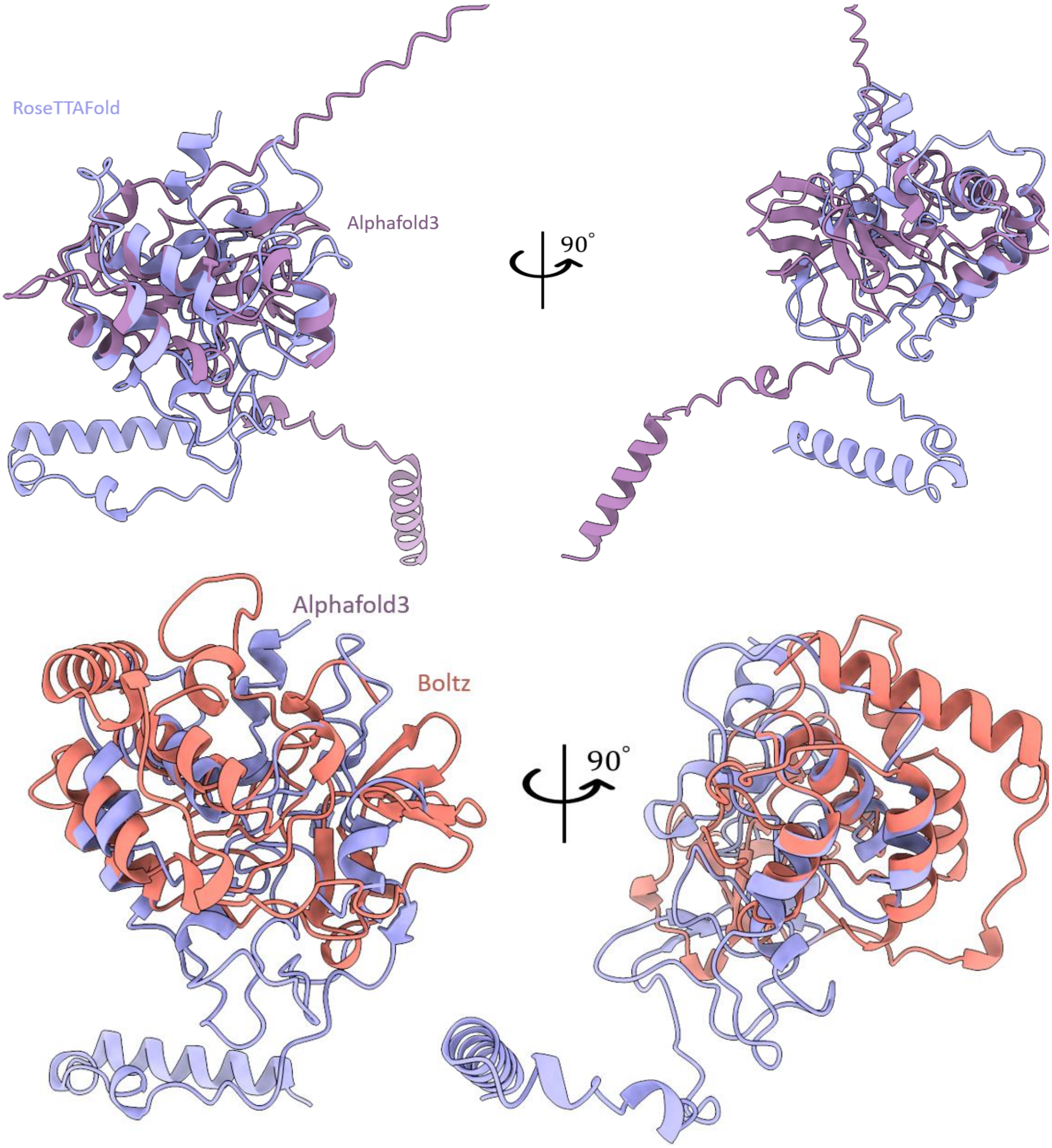

Figure S12. Structural superposition of the AlphaFold3, RoseTTAFold, and Boltz-2 HSTV SP1 models.

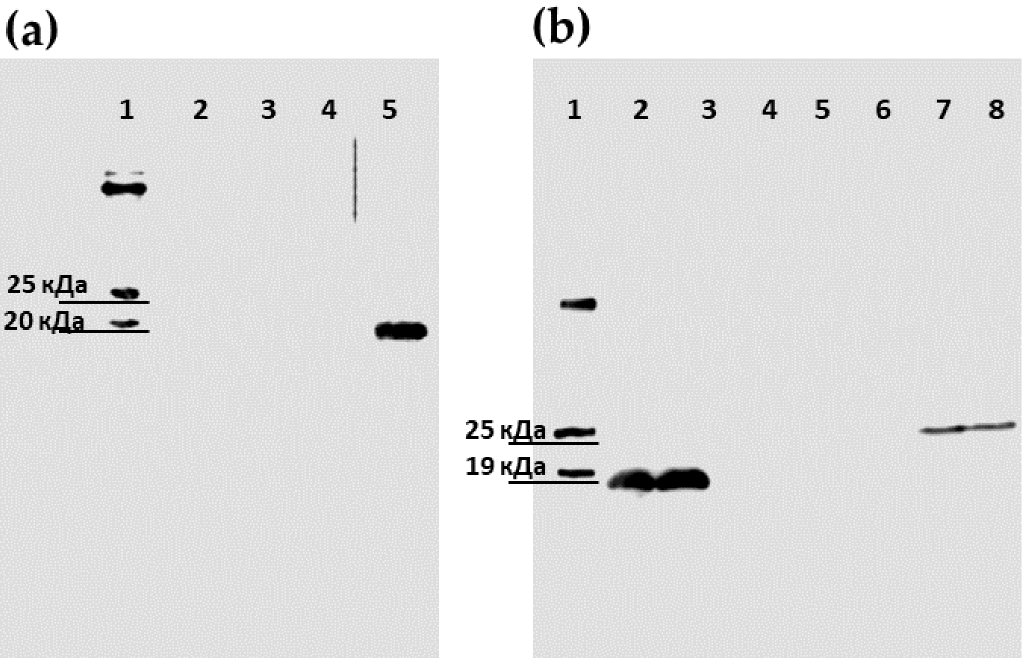

Figure S13. Western-blot results for cell lysate of BL21(DE3)/pET-HSTV-SP1 and KRX/pET-HSTV-SP1 strains:

(a) western blot in non-denaturing conditions: lane 1 – molecular weight ruler, lane 2 – cell lysate of BL21(DE3)/pET-HSTV-SP1, lane 3 – cell lysate of KRX/pET-HSTV-SP1, lane 4 – none proteins, lane 5 – control protein with weight ~19kDa.

(b) western blot in denaturing conditions: lane 1 – molecular weight ruler, lane 2-3 – control protein with weight ~19kDa, lane 4-6 – none proteins, lane 7 – cell lysate of BL21(DE3)/pET-HSTV-SP1, lane 8 – cell lysate of KRX/pET-HSTV-SP1.

(a) Erns, PDB ID: 4DVK | **(b)** Npro, PDB ID: 3ZFN | **(c)** Npro, PDB ID: 4H9K

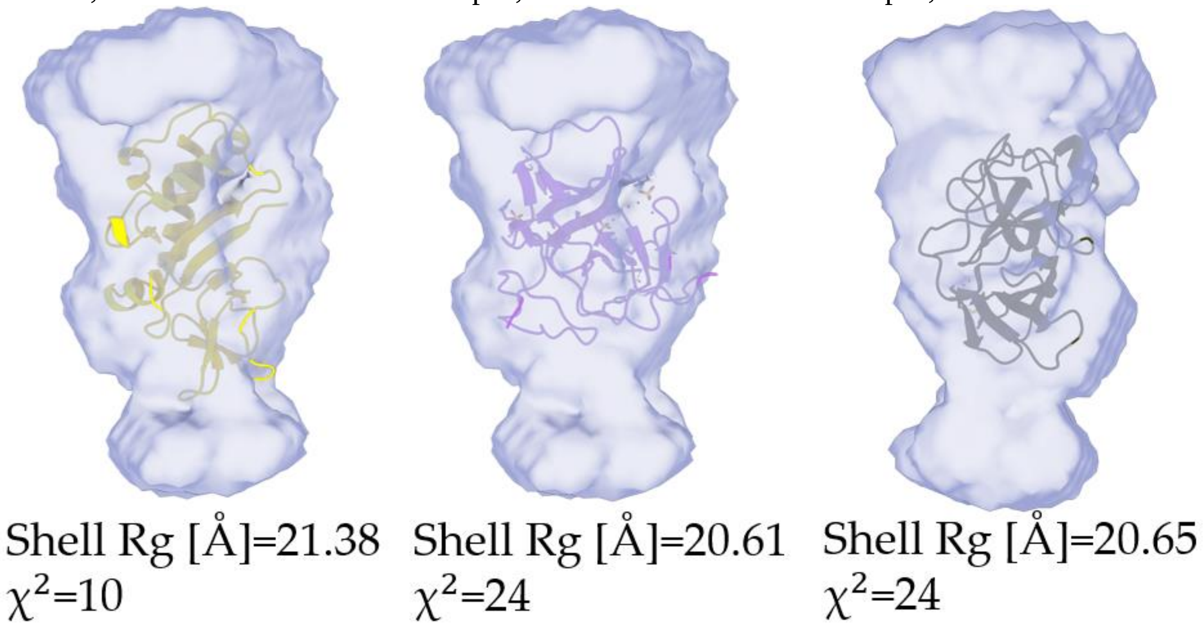

Figure S14. Low-resolution *ab initio* molecular envelopes of HSTV SP1 in solution reconstructed from SAXS using DAMMIF (transparent purple, 3.12 mg/ml data) superimposed with crystal structures of **(a)**Erns, PDB ID: 4DVK, and Npro: **(b)** PDB ID: 3ZFN, **(c)** PDB ID: 4H9K.

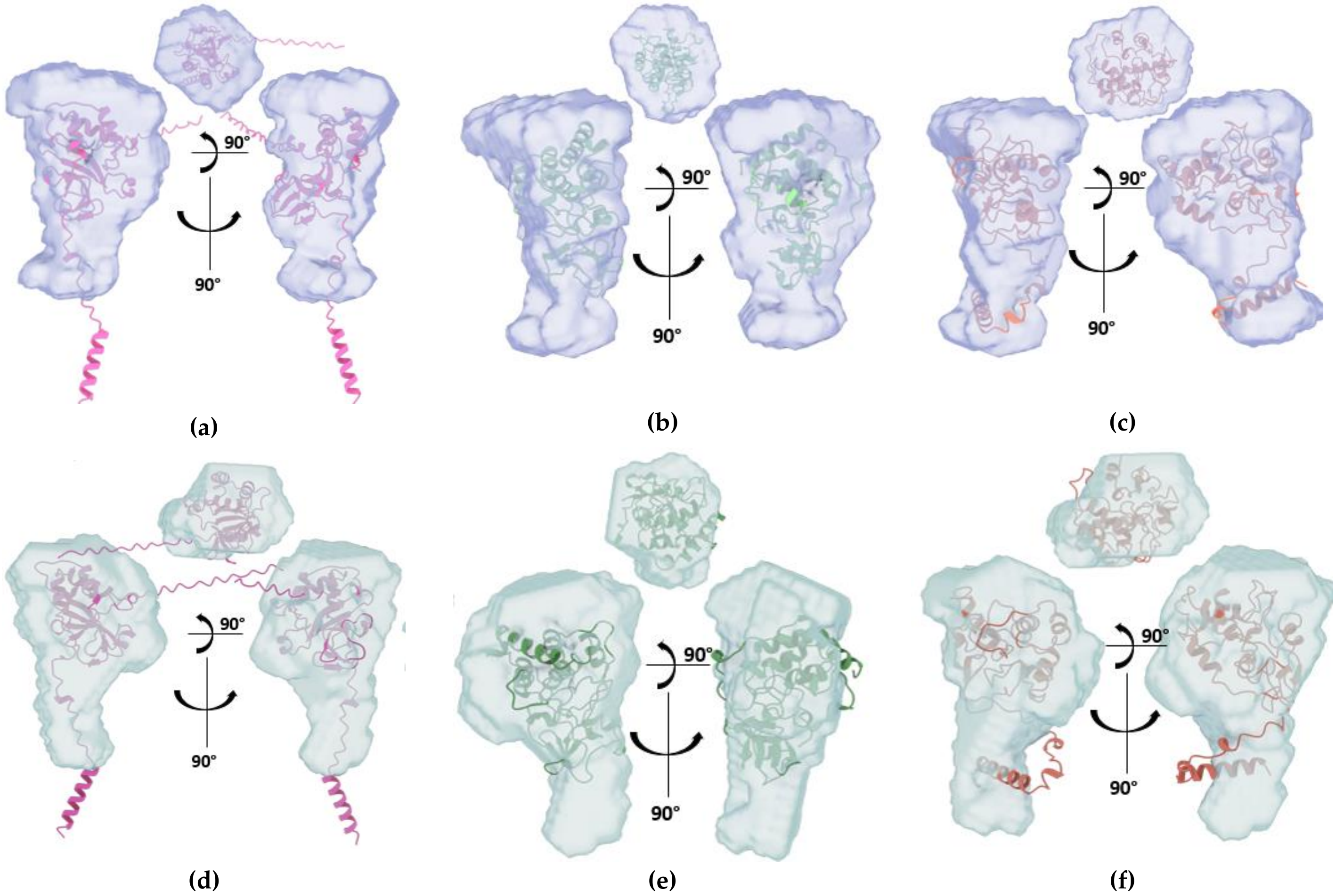

Figure S15. Low-resolution (~26–35 Å) *ab initio* molecular envelopes of HSTV SP1 in solution, re-constructed from SAXS data using DAMMIF at two protein concentrations: 3.12 mg/mL (top, transparent purple envelopes) and 12.31 mg/mL (bottom, transparent green envelopes). **(a, d)** AlphaFold3-predicted HSTV SP1 structural model; **(b, e)** Boltz-2-predicted HSTV SP1 model; **(c, f)** RoseTTAFold-predicted HSTV SP1 model. Each structural model is superimposed onto the corresponding experimental SAXS-derived envelope.
